# Rede nacional de testes moleculares para detecção de *Chlamydia trachomatis* e *Neisseria gonorrhoeae*: experiência de implantação-piloto no Brasil

**DOI:** 10.1590/0102-311XPT123023

**Published:** 2024-07-29

**Authors:** Pâmela Cristina Gaspar, Angélica Espinosa Miranda, Alisson Bigolin, Amanda Alencar Cabral Morais, Mayra Gonçalves Aragón, José Athayde Vasconcelos Morais, José Boullosa Alonso, Leonor Henriette de Lannoy, Mauro Niskier Sanchez, Draurio Barreira Cravo, Ximena Pamela Claudia Díaz Bermúdez, Adele Schwartz Benzaken

**Affiliations:** 1 Departamento de HIV/AIDS, Tuberculose, Hepatites Virais e Infecções Sexualmente Transmissíveis, Ministério da Saúde, Brasília, Brasil.; 2 Programa de Pós-graduação em Saúde Coletiva, Universidade de Brasília, Brasília, Brasil.; 3 Programa de Pós-graduação em Doenças Infecciosas, Universidade Federal do Espírito Santo, Vitória, Brasil.; 4 Instituto Leonidas & Maria Deane, Fundação Oswaldo Cruz, Manaus, Brasil.

**Keywords:** Técnicas de Diagnóstico Molecular, Neisseria gonorrhoeae, Chlamydia trachomatis, Projetos Piloto, Sistema Único de Saúde, Molecular Diagnostic Techniques, Neisseria gonorrhoeae, Chlamydia trachomatis, Pilot Projects, Unified Health System, Técnicas de Diagnóstico Molecular, Neisseria gonorrhoeae, Chlamydia trachomatis, Proyectos Piloto, Sistema Único de Salud

## Abstract

O objetivo deste estudo foi conhecer a opinião dos profissionais participantes da implantação-piloto de testes moleculares para detecção de *Chlamydia trachomatis* e *Neisseria gonorrhoeae* no Sistema Único de Saúde (SUS). Determinou-se a taxa de detecção de *C. trachomatis* e/ou *N. gonorrhoeae* e os fatores associados à infecção. A estratégia contou com laboratórios pertencentes à rede de carga viral de HIV e hepatites virais. A testagem teve como público-alvo pessoas mais vulnerabilizadas às infecções sexualmente transmissíveis, com coleta de amostras de urina e/ou *swabs* vaginal, endocervical e/ou uretral masculino. Questionários foram enviados aos gestores estaduais e profissionais de laboratório sobre a implantação-piloto. De maneira geral, as avaliações foram positivas. Entre as fraquezas, citou-se dificuldades na mudança do processo de trabalho, carência de recursos humanos, pouca sensibilidade de profissionais da assistência e ausência de tubo primário de urina, único insumo não fornecido. Como fortaleza, destaca-se aquisição centralizada de testes, compartilhamento de equipamentos e armazenamento de amostras à temperatura ambiente. Das 16.177 pessoas testadas, 1.004 (6,21%) foram positivas para *C. trachomatis*, 1.036 (6,4%) para *N. gonorrhoeae* e 239 (1,48%) para *C. trachomatis*/*N. gonorrhoeae*. A detecção de infecção ocorreu mais em pessoas jovens (≤ 24 *vs.* > 24 anos) (aOR = 2,65; IC95%: 2,38-2,96), do sexo masculino (aOR = 1,95; IC95%: 1,72-2,21), pardas/pretas (aOR = 1,06; IC95%: 1,05-1,11), na Região Sudeste (aOR = 1,08; IC95%: 1,02-1,13) e em amostras de secreção uretral (aOR = 1,46; IC95%: 1,41-1,52). Os resultados deste estudo demonstraram a importância da disponibilização da testagem em âmbito nacional, os quais subsidiaram a implantação da rede definitiva para detecção de *C. trachomatis*/*N. gonorrhoeae* no SUS.

## Abstract (en)

This study aimed to know the opinion of professionals participating in an experiment to implement a pilot for molecular tests to detect *Chlamydia trachomatis* and *Neisseria gonorrhoeae* at the Brazilian Unified National Health System (SUS). The detection rate of *C. trachomatis* and/or *N. gonorrhoeae* and the factors associated with infection were determined. The strategy included laboratories belonging to the HIV and viral hepatitis viral load network. Testing targeted people who are more vulnerable to sexually transmitted infections and collected urine samples and/or vaginal, endocervical, and/or male urethral swabs. Questionnaires were sent to state managers and laboratory professionals about the implementation of the pilot. Reviews were overall positive. Weaknesses included difficulties changing work processes, lack of human resources, poorly sensitized care professionals, and absence of primary urine tubes, the only input not provided. Strengths included the centralized acquisition of tests, sharing of equipment, and storage of samples at room temperature. Of the 16,177 people who were tested, 1,004 (6.21%) were positive for *C. trachomatis*; 1,036 (6.4%), for *N. gonorrhoeae*; and 239 (1.48%), for *C. trachomatis*/*N. gonorrhoeae* . Detection of any infection occurred more frequently in young people (≤ 24 vs. > 24 years) (adjOR = 2.65; 95%CI: 2.38-2.96), men (adjOR = 1.95; 95%CI: 1.72-2.21), brown/black individuals (adjOR = 1.06; 95%CI: 1.05-1.11), those in Southeastern Brazil (adjOR = 1.08; 95%CI: 1.02-1.13), and in urethral secretion samples (adjOR = 1.46; 95%CI: 1.41-1.52). Results show the importance of making testing available nationwide, which supported the implementation of a definitive network to detection *C. trachomatis*/*N. gonorrhoeae* in SUS.

## Resumen

El objetivo de este estudio fue conocer la opinión de los profesionales participantes de la implantación piloto de pruebas moleculares para la detección de *Chlamydia trachomatis* y *Neisseria gonorrhoeae* en el Sistema Único de Salud brasileño (SUS). Se determinó la tasa de detección de *C. trachomatis* y/o *N. gonorrhoeae* y los factores asociados con la infección. En la estrategia participaron laboratorios pertenecientes a la red de carga viral de VIH y hepatitis virales. La prueba tuvo como público objetivo a personas más vulnerables a las infecciones de transmisión sexual, con recolección de muestras de orina y/o *swabs* vaginal, endocervicales y/o uretral masculino. Se enviaron cuestionarios a los gestores estatales y a los profesionales de laboratorio sobre la implementación piloto. En general, las evaluaciones fueron positivas. Entre las debilidades, se citó las dificultades en el cambio del proceso de trabajo, la falta de recursos humanos, los profesionales de la asistencia poco sensibilizados y la ausencia del contenedor de orina primaria, el único insumo no suministrado. Como fortalezas, se destaca la adquisición centralizada de pruebas, el intercambio de equipos y el almacenamiento de muestras a temperatura ambiente. De las 16.177 personas evaluadas, 1.004 (6,21%) fueron positivas para *C. trachomatis*, 1.036 (6,4%) para *N. gonorrhoeae* y 239 (1,48%) para *C. trachomatis*/*N. gonorrhoeae*. La detección de alguna infección ocurrió más en personas jóvenes (≤ 24 *vs.* > 24 años) (aOR = 2,65; IC95%: 2,38-2,96), del sexo masculino (aOR = 1,95; IC95%: 1,72-2,21), parda/negra (aOR = 1,06; IC95%: 1,05-1,11), localizadas en la región Sudeste (aOR = 1,08; IC95%: 1,02-1,13) y en muestras de secreción uretral (aOR = 1,46; IC95%: 1,41-1,52). Los resultados de este estudio demostraron la importancia de la disponibilidad de la prueba a nivel nacional, los cuales subsidiaron la implantación de la red definitiva para detección de *C. trachomatis*/*N. gonorrhoeae* en el SUS.

## Introdução

As infecções sexualmente transmissíveis (IST) são responsáveis por altas taxas de morbidade e por elevados custos em saúde pública ^1^. Entre as IST, destacam-se as infecções causadas por *Chlamydia trachomatis* e *Neisseria gonorrhoeae* que podem gerar danos graves e até irreversíveis à saúde, tais como infertilidade e doença inflamatória pélvica (DIP), implicando graves consequências médicas, sociais, psicológicas e econômicas ^2^ ^,^ ^3^. Adicionalmente, casos não tratados de maneira adequada podem levar à seleção de linhagens resistentes a antimicrobianos, com impacto negativo nas opções de tratamento disponíveis ^4^ ^,^ ^5^ ^,^ ^6^. Nos homens, as infecções por *C. trachomatis* e *N. gonorrhoeae* são predominantemente sintomáticas, causando a síndrome do corrimento uretral, enquanto nas mulheres essas infecções se mostram na maioria das vezes assintomáticas ^2^ ^,^ ^3^. Estratégias de rastreamento são essenciais para reduzir a morbidade associada à infecção por meio do diagnóstico oportuno e do tratamento precoce, sobretudo nas populações mais vulnerabilizadas às IST ^3^.

Para a investigação de infecções causadas por *C. trachomatis* e/ou *N. gonorrhoeae*, tanto nos casos sintomáticos como nos assintomáticos, constituem método de escolha os testes de biologia molecular, como os testes de amplificação do ácido nucleico (NAAT), sendo o teste de reação em cadeia da polimerase (PCR), o mais comum deles ^7^ ^,^ ^8^. Esses testes estão incorporados ao Sistema Único de Saúde (SUS) desde março de 2018 ^9^; no entanto, sua oferta ocorria de forma esporádica, principalmente associada a serviços de profilaxia pré-exposição (PrEP) ao HIV ^3^. Nesse contexto, uma vez que já existia uma rede nacional consolidada de laboratórios que realizavam testes automatizados de quantificação da carga viral de HIV e hepatites virais B e C, gerenciada pelo Ministério da Saúde, agregou-se a essa rede a implantação-piloto de testes de biologia molecular para detecção de *C. trachomatis* e *N. gonorrhoeae*, visando a convergência tecnológica para a ampla oferta de testagem.

Dessa forma, o objetivo deste estudo foi conhecer a opinião de profissionais gestores atuantes na coordenação estadual de IST, e de profissionais dos laboratórios responsáveis localmente pela implantação-piloto dos testes de biologia molecular para detecção de *C. trachomatis* e *N. gonorrhoeae* no âmbito do SUS sobre as fortalezas e fragilidades do processo; determinar a proporção de *C. trachomatis* e/ou *N. gonorrhoeae* encontrada; e analisar os fatores associados à maior proporção de infecção entre as pessoas que foram testadas.

## Metodologia

### Tipo do estudo e população do estudo

Trata-se de estudo do tipo descritivo, conduzido entre 2021 e 2022, envolvendo pessoas que participaram da implantação-piloto dos testes de biologia molecular para detecção de *C. trachomatis* e *N. gonorrhoeae* no âmbito do SUS, nas 27 Unidades da Federação (UF), sendo elas profissionais gestores atuantes na coordenação estadual de IST, profissionais de laboratório responsáveis localmente pela implantação-piloto e pacientes. Os testes da implantação-piloto tiveram como foco as seguintes populações com maior vulnerabilidade às IST, conforme previsto no Protocolo Clínico e Diretrizes Terapêuticas para Atenção Integral às Pessoas com Infecções Sexualmente Transmissíveis (PCDT-IST) ^3^: pessoas em PrEP, pessoas vivendo com HIV/aids (PVHA) no momento do diagnóstico, pessoas atendidas em serviços de referência para IST e gestantes em situação de maior vulnerabilidade às IST, de acordo com avaliação clínico-epidemiológica.

Em um primeiro momento, realizou-se a análise dos dados secundários provenientes dos questionários aplicados pelo Ministério da Saúde aos profissionais gestores atuantes na coordenação estadual de IST, e de profissionais dos laboratórios responsáveis localmente pela implantação-piloto para conhecer a opinião dos entrevistados sobre as fortalezas e fragilidades do processo. Na segunda etapa, determinou-se a proporção das infecções por *C. trachomatis* e/ou *N. gonorrhoeae* entre as pessoas que foram testadas na rede-piloto e realizou-se a análise dos fatores associados à presença da infecção por *C. trachomatis* e/ou *N. gonorrhoeae* nessa população.

### Descrição da rede-piloto

A rede-piloto da implantação dos testes de biologia molecular para detecção de *C. trachomatis* e *N. gonorrhoeae* foi composta por 34 laboratórios, indicados pelas coordenações estaduais de IST, sendo, pelo menos, um laboratório por estado. Esses laboratórios deveriam necessariamente fazer parte da Rede Nacional de Laboratórios para Quantificação da Carga Viral do HIV e Hepatites Virais B e C, visando o aproveitamento da capacidade local já instalada para a execução de testes de biologia molecular.

O Ministério da Saúde, por meio do Departamento de HIV/Aids, Tuberculose, Hepatites Virais e Infecções Sexualmente Transmissíveis, coordenou a implantação-piloto, com a gestão de todos os processos, no que tange à articulação com as coordenações estaduais e laboratórios, produção de material informativo e notas técnicas, organização de capacitações, realização de *webinars*, e aquisição de todos os insumos para detecção de ácidos nucleicos de *C. trachomatis* e *N. gonorrhoeae* compatíveis com a plataforma automatizada (Abbott RealTime; https://www.molecular.abbott) então existente, compondo a Rede de Biologia Molecular para a Quantificação da Carga Viral de HIV e Hepatites Virais B e C. O *kit* de coleta Abbott multi-Collect Specimen Collection utilizado continha um tubo de transporte com tampa, com 1,2mL de tampão de transporte de espécime, um *swab* de coleta de espécime estéril e uma pipeta de transferência de urina descartável. Tubos primários para coleta de urina não faziam parte do *kit*, ficando a cargo dos serviços locais. As amostras realizadas na implantação-piloto foram: *swab* vaginal, *swab* endocervical, *swab* uretral masculino e urina.

O treinamento de equipes e a implantação da rede contaram com a publicação de documentos orientadores e a realização de capacitações virtuais e presenciais para gestores, profissionais de laboratório e profissionais clínicos. Para tanto, realizou-se o envio de documentos oficiais com informações gerais sobre a rede-piloto e foram organizadas reuniões *online* com gestores, profissionais de laboratório e profissionais dos serviços de assistência. O Ministério da Saúde também promoveu capacitações *online* sobre coleta de amostras, com disponibilização dos vídeos para posterior visualização de livre acesso. O fabricante do teste realizou treinamentos *online* e presenciais para todos os profissionais dos laboratórios participantes da rede-piloto, com fornecimento de instruções de uso em português brasileiro. Por fim, um *webinar* sobre a oferta da testagem no SUS foi promovido pelo Ministério da Saúde, em parceria com a Sociedade Brasileira de Doenças Sexualmente Transmissíveis (SBDST), para divulgar, orientar e estimular os profissionais de saúde clínicos a solicitarem testagem.

Para envio dos insumos aos locais, utilizou-se a mesma cadeia logística existente para os demais testes de quantificação da carga viral de HIV e hepatites virais B e C, com envio dos kits de coleta e insumos dos testes pela empresa fornecedora diretamente aos laboratórios pertencentes à rede-piloto. As entregas de insumos aos locais ocorreram mensalmente, uma vez que não havia espaço suficiente nos laboratórios para armazenamento de grande quantidade de materiais em temperatura ambiente e sob refrigeração.

A configuração da rede local de serviços de coleta ficou a critério de cada UF, podendo abranger uma ou mais unidades de atenção à saúde localizadas na capital e/ou no interior. Cada UF ficou responsável pela descentralização dos kits de coleta do laboratório para os serviços de atenção à saúde e pela logística de transporte de amostras até o laboratório.

As amostras vaginais, endocervicais e uretrais foram coletadas por profissionais de saúde com auxílio de *swabs* e colocadas dentro do tubo de transporte, podendo ficar armazenadas por até 14 dias em temperatura ambiente, e por até 90 dias a ≤ -10ºC. As amostras de urina foram coletadas em tubo primário (não estéril) e, em seguida, transferidas para o tubo de transporte em quantidade suficiente, conforme indicado na janela de volumes mínimo e máximo no tubo. As condições de armazenamento foram as mesmas para tubos de urina e tubos contendo *swabs*.

As amostras foram cadastradas no sistema Gerenciador de Ambiente Laboratorial (GAL; http://gal.datasus.gov.br/GALL/index.php) pelos serviços no local da coleta. Entre as informações inseridas no sistema, destacam-se: unidade solicitante do exame, data de nascimento, idade, sexo, raça/cor do paciente, data de coleta, tipo de material biológico e nome do exame. Após transportadas aos laboratórios, a data de recebimento da amostra e o nome do laboratório executor também eram registrados no GAL, bem como a data de liberação do resultado das análises, discriminando-se entre detecção de *C. trachomatis*, *N. gonorrhoeae* ou ambos. O tempo ideal preconizado entre o recebimento da amostra e a liberação do resultado pelo laboratório foi de até 15 dias. Esse período foi determinado com base no tempo preconizado como ideal para a liberação do resultado dos exames que compõem a Rede Nacional de Laboratórios para Quantificação da Carga Viral de HIV e Hepatites Virais B e C.

### Questionários sobre o processo da implantação-piloto

O Ministério da Saúde aplicou questionários aos profissionais gestores atuantes na coordenação estadual de IST e aos profissionais dos laboratórios responsáveis localmente pela implantação-piloto, com o objetivo de conhecer a opinião desses atores sobre o processo e identificar pontos que necessitam de correção, considerando a expansão das atividades no âmbito do SUS. Não houve limite no número de profissionais respondentes por UF ou por laboratório. Foram utilizados dois questionários distintos previamente validados pelo Ministério da Saúde, sendo um para os profissionais gestores estaduais e outro para os profissionais dos laboratórios, ambos contendo perguntas sobre as atividades locais, a opinião sobre os materiais que orientaram a implantação-piloto e os treinamentos, bem como as fragilidades e fortalezas do processo. Esses questionários foram aplicados no período de outubro a dezembro de 2022, de forma *online*, com auxílio de um sistema do Ministério da Saúde denominado Inquérito. Existiam questões em que os profissionais podiam escolher mais de uma alternativa e outras com possibilidade de selecionar apenas uma. Os dados secundários provenientes das respostas dos questionários foram disponibilizados pelo Ministério da Saúde de forma anônima. Os resultados são apresentados conforme a frequência de profissionais que escolheram as alternativas para cada questão.

###  Determinação da proporção de detecção de *C. trachomatis* e/ou *N. gonorrhoeae* e análise de fatores associados à maior detecção 

Os dados secundários referentes aos resultados das testagens cadastradas no GAL como “Pesquisa de Multipatógenos IST” foram disponibilizados pelo Ministério da Saúde de forma anônima, atribuindo-se um código para cada pessoa testada no âmbito da implantação-piloto. Determinou-se a proporção de detecção de infecção por *C. trachomatis* e/ou *N. gonorrhoeae* e de coinfecção *C. trachomatis* e *N. gonorrhoeae* nessa população. Os dados categóricos são apresentados de forma descritiva, por meio de frequência. Variáveis numéricas são apresentadas por meio de média, mediana e desvio padrão. Na análise bivariada foi calculado teste qui-quadrado para identificação de fatores associados à presença de infecção por *C. trachomatis* e/ou *N. gonorrhoeae*. Realizou-se a regressão logística para análise de fatores associados à maior detecção de alguma infecção com relação a idade, raça/cor, tipo de amostra biológica e região geográfica. O programa SPSS, versão 25.0 (https://www.ibm.com/), foi utilizado para conduzir as análises estatísticas.

### Aspectos éticos

O estudo foi aprovado pelo Comitê de Ética da Faculdade de Ciências da Saúde da Universidade de Brasília (UnB; nº 5.655.475, CAAE: 62249722.5.0000.0030) e pela Comissão Nacional de Ética em Pesquisa (CONEP; nº 5.864.830, CAAE: 62249722.5.3001.0008).

## Resultados

Entre os meses de outubro de 2021 e dezembro de 2022 foram realizados 16.177 testes de *C. trachomatis*/*N. gonorrhoeae* com resultados liberados no GAL, os quais foram executados no âmbito da rede-piloto. Todas as 27 UF tiveram laboratórios integrando a rede-piloto, os quais foram capacitados e estavam aptos a receber os insumos. Houve registro de solicitação do exame por 93 municípios, distribuídos em 20 UF, com uma quantidade distinta de resultados liberados por UF, variando de nove, no Pará, a 3.365, em Santa Catarina, e uma média de 809 amostras por UF. Mensalmente, a rede-piloto executou em média 1.078 testes, com tempo médio de 18 dias para a liberação do resultado e uma mediana de 10 dias, variando de 0 a 144 dias.

Não foram localizadas no GAL solicitações de testes de *C. trachomatis* e *N. gonorrhoeae* de sete UF, possivelmente porque essas UF têm sistemas próprios e não usam o GAL, porque cadastraram o teste em outro exame, ou então porque não houve solicitação ou coleta local de amostras, embora a gestão e o laboratório integrassem a rede-piloto.

### Questionários sobre o processo da implantação-piloto: gestão estadual

Um total de 30 profissionais gestores atuantes na coordenação estadual de IST de todas as UF do Brasil responderam ao questionário aplicado pelo Ministério da Saúde. Quando indagados sobre quais ações são desenvolvidas pela gestão estadual para o enfrentamento das IST causadas por *C. trachomatis* e *N. gonorrhoeae*, 100% relataram distribuir os preservativos enviados pela esfera federal e 67% afirmaram promover capacitações aos profissionais da saúde sobre os protocolos de manejo clínico de IST. Apenas 20% reportaram a aquisição própria de testes de biologia molecular para detecção de *C. trachomatis* e *N. gonorrhoeae*, apesar de esses testes estarem incorporados ao SUS desde 2018.

Sobre o processo de implantação da rede-piloto, 90% dos respondentes qualificaram como excelente/boa a condução do processo; 90% avaliaram como excelentes/bons os materiais de apoio (*e-mails*, ofício, protocolos de uso dos sistemas, protocolo de tratamento) fornecidos; e 77% classificaram como excelente/bom o *webinar* de capacitação sobre coleta de amostras biológicas conduzido pelo Ministério da Saúde e pela empresa fabricante, embora cerca de 17% informaram não ter assistido ao *webinar* da capacitação. Ainda, foi realizado um webinar sobre a importância da biologia molecular para detecção de *C. trachomatis* e *N. gonorrhoeae* no manejo clínico, o qual foi avaliado pela maioria dos gestores como excelente/bom (77%), sendo que 20% alegaram não tê-lo acompanhado.

Quando questionados sobre o número de serviços que fazem parte da rede estadual como ponto de coleta, 20% informaram um serviço, 23% de dois a quatro serviços, 33% de cinco a oito serviços e 20% apontaram nove ou mais serviços. Entre os tipos de serviço incluídos na rede-piloto como sítio de coleta, os mais citados foram: Serviço de Assistência Especializada (SAE) (80%), Centro de Testagem e Aconselhamento (CTA) (63%), Centro de Referência em IST/Aids (47%) e Unidade de Atenção Primária à Saúde (40%). Esses dados permitem conhecer como é a capilaridade do acesso aos testes de biologia molecular no território.

A Tabela 1 apresenta as opiniões dos gestores sobre as principais fragilidades relacionadas à rede-piloto, divididas em duas categorias: local/recursos humanos e infraestrutura/características do teste; a Tabela 1 também mostra as principais fortalezas do processo de implantação da nova rotina relacionada à testagem de biologia molecular para detecção de *C. trachomatis* e *N. gonorrhoeae*. Observa-se, portanto, que as principais fragilidades reportadas por mais da metade dos gestores estiveram relacionadas à necessidade de implantação de um novo fluxo local (envio de *kit* de coleta para os serviços e transporte das amostras até o laboratório), à carência de recursos humanos, à sobrecarga de demandas nos serviços de saúde e à falta de infraestrutura para a coleta de amostras biológicas femininas (vaginais e cervicais) e de secreção uretral. Sobre as fortalezas, exceto para a alternativa relacionada à sensibilização dos profissionais da assistência sobre a importância dos testes para a detecção de *C. trachomatis* e *N. gonorrhoeae*, todas as demais alternativas foram assinaladas por mais da metade dos profissionais, a exemplo da gestão centralizada dos insumos, do compartilhamento de equipamentos, da possibilidade de armazenamento das amostras em temperatura ambiente por longos períodos e da detecção de mais de um tipo de patógeno em uma mesma amostra.

**Tabela 1:** Fragilidades e fortalezas no processo de implantação da nova rotina relacionada à testagem de biologia molecular para detecção de *Chlamydia trachomatis* e *Neisseria gonorrhoeae*, segundo os gestores da vigilância estadual de infecções sexualmente transmissíveis (IST).

	Respondentes % (n) *
Principais fragilidades relacionadas à rede local/recursos humanos no processo de implantação da nova rotina relacionada à testagem de biologia molecular para detecção de *C. trachomatis* e *N. gonorrhoeae*	
Dificuldade de estabelecimento de novo fluxo dentro da rede de atenção à saúde	67 (20)
Carência de recursos humanos nos serviços de assistência à saúde	57 (17)
Sobrecarga de demandas nos serviços de assistência à saúde	53 (16)
Falta de interesse dos profissionais clínicos em realizar a solicitação deste teste	43 (13)
Carência de recursos humanos na gestão estadual	37 (11)
Sobrecarga de demandas na gestão estadual	33 (10)
Dificuldade de captação de pacientes	27 (8)
Dificuldade de capacitação dos profissionais de coleta	20 (6)
Carência de recursos humanos nos laboratórios executores dos testes	17 (5)
Sobrecarga de demandas nos laboratórios executores dos testes	13 (4)
Dificuldade de capacitação dos profissionais de laboratório	3 (1)
Outros	23 (7)
Principais fragilidades relacionadas à infraestrutura/características do teste no processo de implantação da nova rotina relacionada à testagem de biologia molecular para detecção de *C. trachomatis* e *N. gonorrhoeae*	
Carência de infraestrutura nos serviços de assistência à saúde para coleta de amostra de conteúdo vaginal e cervical	63 (19)
Carência de infraestrutura nos serviços de assistência à saúde para coleta de amostra de secreção uretral	57 (17)
Dificuldade na logística do transporte das amostras coletadas para o laboratório	37 (11)
Impossibilidade de análise de amostras anais (extragenitais) pelo teste utilizado na implantação-piloto	30 (9)
Não fornecimento dos tubos primários de coleta de amostra de urina juntamente com os demais itens do kit de coleta de amostras	27 (8)
Impossibilidade de análise de amostras da orofaringe (extragenital) pelo teste utilizado na implantação-piloto	23 (7)
Dificuldade na logística do transporte dos kits de coleta para o serviço	23 (7)
Carência de infraestrutura nos serviços de assistência à saúde para coleta de amostra de urina	20 (6)
Carência de infraestrutura nos serviços de assistência à saúde para armazenamento da amostra	20 (6)
Dificuldade de armazenamento das amostras coletadas no serviço	20 (6)
Outros	10 (3)
Principais fortalezas no processo de implantação da nova rotina relacionada à testagem de biologia molecular para detecção de *C. trachomatis* e *N. gonorrhoeae*	
Aquisição e distribuição dos testes de forma centralizada pelo Ministério da Saúde	87 (26)
Compartilhamento dos mesmos equipamentos da Rede de Carga Viral de HIV e Hepatites Virais	87 (26)
Possibilidade de armazenamento da amostra em temperatura ambiente por 14 dias	87 (26)
Possibilidade de utilizar diferentes tipos de amostras biológicas para investigação de *C. trachomatis* e *N. gonorrhoeae* (urina, esfregaço endocervical, esfregaço vaginal e esfregaço uretral)	80 (24)
Possibilidade de investigação do agente infeccioso causador das síndromes, mesmo que o tratamento inicial tenha ocorrido de forma sindrômica	70 (21)
Possibilidade de detecção de infecção mesmo nos casos assintomáticos	67 (20)
Possibilidade de armazenamento da amostra por 90 dias sob congelamento	60 (18)
Sensibilização dos profissionais de saúde sobre a importância da testagem para detecção de *C. trachomatis* e *N. gonorrhoeae*	43 (13)
Outros	7 (2)

Fonte: questionário de avaliação da rede-piloto de testes de biologia molecular para detecção de *C. trachomatis* e *N. gonorrhoeae* no âmbito do Sistema Único de Saúde (SUS).

* Frequência de profissionais que assinalaram cada alternativa. Mais de uma alternativa poderia ser assinalada por profissional, portanto, a soma das alternativas é superior a 100%

### Questionários sobre o processo da implantação-piloto: laboratório

O questionário destinado aos laboratórios foi respondido por profissionais pertencentes a 33 laboratórios participantes da rede-piloto, não havendo representação, portanto, de apenas um laboratório. A maioria dos respondentes desempenha a função de analista de nível superior (81%). Quando questionados sobre o treinamento para a execução dos testes, 92% afirmaram que a carga horária total disponibilizada para a capacitação foi suficiente, bem como o conteúdo apresentado (89%). Cerca de 57% alegaram não ter utilizado a plataforma educacional *online* disponibilizada pela empresa fabricante dos testes como ferramenta de apoio.

No que diz respeito à logística dos insumos, 76% dos respondentes não tiveram dificuldade de preencher o sistema logístico. O fabricante enviou todos os itens aos laboratórios, tanto os reagentes dos kits de análise como os materiais para coleta, que foram distribuídos pelos laboratórios aos pontos de coleta. No que diz respeito a esse processo, 33% dos participantes mencionaram a inexistência prévia de um fluxo estabelecido para o envio de insumos de coleta de amostras biológicas aos serviços de saúde pelo laboratório executor, o que se caracterizou como um fator dificultador. Isso porque, comumente, é de responsabilidade da gestão o envio desse tipo de insumo aos serviços de assistência. Ainda sobre esse tema, como o kit de coleta não continha o tubo primário para a coleta de urina (somente o tubo de conservação), questionou-se a não distribuição desse tubo pelo laboratório aos serviços. Cerca de 65% dos profissionais alegaram que as unidades de saúde já possuíam esse tipo de material no local, e 22% afirmaram que o tubo primário deveria estar contido no kit de coleta do fabricante.

Sobre a principal dificuldade no processo de implantação da rede de biologia molecular para detecção de *C. trachomatis* e *N. gonorrhoeae* no território, na visão dos laboratórios, 51% assinalaram que os profissionais da assistência estavam pouco sensibilizados sobre a importância da testagem e, portanto, não realizavam a solicitação dos testes, apesar da sua disponibilidade na rede. Do total de profissionais de laboratórios respondentes, 89% alegaram conseguir implementar os exames de *C. trachomatis* e *N. gonorrhoeae* na rotina, e 76% afirmaram que a inclusão de mais um teste não impactou a rotina dos demais exames (quantificação da carga viral de HIV e hepatites virais B e C), os quais compartilhavam o mesmo equipamento. As intercorrências com amostras biológicas estiveram mais frequentemente relacionadas ao volume de urina no tubo, seja em excesso (22%), ou em quantidade insuficiente (22%).

###  Proporção de detecção de *C. trachomatis* e/ou *N. gonorrhoeae* e análise de fatores associados à maior proporção de detecção 

Do total de 16.177 pessoas que realizaram os testes no âmbito da rede-piloto, a média de idade foi de 33,1 (± 10,6) anos, e a mediana, de 31,0 anos. A maioria (68,6%) se declarou do sexo masculino e de raça/cor branca (37,7%), tendo realizado a coleta do tipo urina (57%) e em serviços localizados na Região Sudeste (43,8%).

A infecção por *C. trachomatis* e/ou *N. gonorrhoeae* foi detectada em 1.828 (11,3%) amostras, sendo que em 1.004 (6,2%) houve detecção de *C. trachomatis*, em 1.036 (6,4%) de *N. gonorrhoeae* e em 239 (1,5%) de *C. trachomatis* e *N. gonorrhoeae* (coinfecção). As proporções de detecção para *C. trachomatis* e *N. gonorrhoeae*, respectivamente, foram de 6,5% e 1,6% em mulheres e de 6,1% e 8,6% em homens. A Tabela 2 apresenta os resultados das testagens de acordo com as características sociodemográficas.

**Tabela 2:** Características sociodemográficas e resultado dos testes para detecção de *Chlamydia trachomatis* e *Neisseria gonorrhoeae* por biologia molecular executados na estratégia de implantação-piloto.

Características	Amostra	***C. trachomatis* não detectado/** ***N. gonorrhoeae* não detectado**	***C. trachomatis* detectado**	***N. gonorrhoeae* detectado**	***C. trachomatis* detectado/** ***N. gonorrhoeae* detectado**
	n (%)	n (%)	n (%)	n (%)	n (%)
Idade (anos)					
≤ 14	180 (1,11)	167 (1,16)	7 (0,70)	7 (0,68)	1 (0,42)
15-19	603 (3,73)	436 (3,04)	113 (11,25)	84 (8,11)	33 (13,81)
20-24	2.530 (15,64)	2.017 (14,06)	292 (29,08)	302 (29,15)	88 (36,82)
25-40	9.476 (58,58)	8.544 (59,54)	489 (48,71)	531 (51,25)	103 (43,10)
41-88	3.388 (20,94)	3.185 (22,20)	103 (10,26)	112 (10,81)	14 (5,86)
Total	16.177 (100,00)	14.349 (100,00)	1.004 (100,00)	1.036 (100,00)	239 (100,00)
Sexo					
Feminino	5.081 (31,41)	4.688 (32,67)	331 (32,97)	82 (7,92)	31 (12,97)
Masculino	11.096 (68,59)	9.661 (67,33)	673 (67,03)	954 (92,08)	208 (87,03)
Total	16.177 (100,00)	14.349 (100,00)	1.004 (100,00)	1.036 (100,00)	239 (100,00)
Raça/Cor					
Amarela	1.916 (11,84)	1.653 (11,52)	152 (15,14)	145 (14,00)	39 (16,32)
Branca	6.093 (37,66)	5.590 (38,96)	303 (30,18)	245 (23,65)	60 (25,10)
Indígena	16 (0,10)	13 (0,09)	2 (0,20)	1 (0,10)	0
Parda/Preta	4.144 (25,62)	3.585 (24,98)	317 (31,57)	313 (30,21)	75 (31,38)
Não declarado	4.008 (24,78)	3.508 (24,45)	230 (22,91)	332 (32,05)	65 (27,20)
Total	16.177 (100,00)	14.349 (100,00)	1.004 (100,00)	1.036 (100,00)	239 (100,00)
Tipo de amostra					
Secreção endocervical	693 (4,28)	646 (4,50)	36 (3,59)	17 (1,64)	6 (2,51)
Secreção vaginal	1.206 (7,46)	1.106 (7,71)	92 (9,16)	17 (1,64)	9 (3,77)
Urina (mulher)	2.973 (18,38)	2.733 (19,05)	198 (19,72)	48 (4,63)	16 (6,69)
Secreção uretral	1.949 (12,05)	1.250 (8,71)	289 (28,78)	535 (51,64)	129 (53,97)
Urina (homem)	9.216 (56,97)	8.496 (59,21)	376 (37,45)	409 (39,48)	78 (32,64)
Outro (extragenital) *	140 (0,87)	118 (0,82)	13 (1,29)	10 (0,97)	1 (0,42)
Total	16.177 (100,00)	14.349 (100,00)	1.004 (100,00)	1.036 (100,00)	239 (100,00)
Região geográfica					
Norte	895 (5,53)	750 (5,23)	24 (2,39)	5 (0,48)	2 (0,84)
Nordeste	2.294 (14,18)	1.858 (12,95)	217 (21,61)	282 (27,22)	65 (27,20)
Centro-oeste	866 (5,35)	865 (6,03)	58 (5,78)	70 (6,76)	12 (5,02)
Sudeste	7.084 (43,79)	6.124 (42,68)	501 (49,90)	592 (57,14)	136 (56,90)
Sul	5.038 (31,14)	4.752 (33,12)	204 (20,32)	87 (8,40)	24 (10,04)
Total	16.177 (100,00)	14.349 (100,00)	1.004 (100,00)	1.036 (100,00)	239 (100,00)

Fonte: sistema Gerenciador de Ambiente Laboratorial (GAL).

* Tipo de amostras biológicas não validadas para o teste, de acordo com o fabricante.

Na análise sobre possíveis fatores associados à presença de alguma infecção (*C. trachomatis* e/ou *N. gonorrhoeae*) entre as pessoas que foram testadas no âmbito da implantação-piloto, encontramos que os testes positivos ocorreram mais em pessoas jovens (≤ 24 *vs.* >24 anos) (*odds ratio* ajustada - aOR = 2,65; intervalo de 95% de confiança - IC95%: 2,38-2,96), do sexo masculino (aOR = 1,95; IC95%: 1,72-2,21), parda/preta (aOR = 1,06; IC95%: 1,05-1,11), e estavam localizadas na Região Sudeste (aOR = 1,08; IC95%: 1,02-1,13). *C. trachomatis* e/ou *N. gonorrhoeae* foi mais detectado em amostras de secreção uretral (aOR = 1,46; IC95%: 1,41-1,52) do que nas demais amostras analisadas (Tabela 3).

**Tabela 3:** Análise dos fatores associados à maior proporção de infecção por *Chlamydia trachomatis* e/ou *Neisseria gonorrhoeae* nas amostras das pessoas testadas no âmbito da estratégia de implantação piloto dos testes.

Características	Total	Pelo menos uma infecção	OR bruta (IC95%)	OR ajustada (IC95%)
	n (%)	n (%)		
Idade (anos)			p < 0,001	
≤ 24	3.313 (20,5)	693 (37,9)	2,7 (2,5-3,0)	2,65 (2,38-2,96)
> 24	12.864 (79,5)	1.135 (62,1)	Referência	
Total	16.177 (100,00)	1.828 (100,00)		
Sexo			p < 0,001	
Feminino	5.081 (31,4)	393 (21,5)	Referência	
Masculino	11.096 (68,6)	1.435 (78,5)	1,8 (1,6-2,0)	1,95 (1,72-2,21)
Total	16.177 (100,00)	1.828 (100,00)		
Raça/Cor			p = 0,006	
Parda/Preta	4.144 (25,6)	559 (30,6)	1,28 (1,11-1,47)	1,06 (1,05-1,11)
Amarela	1.916 (11,8)	263 (14,4)	1,25 (1,04-1,49)	
Indígena	16 (0,1)	3 (0,2)	2,50 (0,64-9,71)	
Não declarado	4.008 (24,8)	500 (27,3)	1,09 (0,94-1,25)	
Branca	6.093 (37,7)	503 (27,5)	Referência	
Total	16.177 (100,00)	1.828 (100,00)		
Tipo de amostra			p < 0,001	
Secreção uretral masculina	1.949 (12,0)	699 (38,2)	7,09 (5,41-11,11)	1,46 (1,41-1,52)
Secreção endocervical	693 (4,3)	47 (2,6)	0,63 (0,46-0,88)	
Secreção vaginal	1.206 (7,5)	100 (5,5)	1,28 (0,13-1,70)	
Outro	140 (0,9)	22 (1,2)	1,90 (0,92-3,07)	
Urina (mulher)	2.973 (18,4)	240 (13,1)	0,93 (0,80-1,09)	
Urina (homem)	9.216 (57,0)	720 (39,4)	Referência	
Total	16.177 (100,00)	1.828 (100,00)		
Região geográfica			p < 0,001	
Norte	895 (5,5)	30 (1,6)	0,57 (0,38-0,84)	
Nordeste	2.294 (14,2)	436 (23,9)	1,06 (0,85-1,32)	
Centro-oeste	866 (5,4)	116 (6,4)	0,47 (0,36-0,62)	
Sudeste	7.084 (43,8)	960 (52,5)	2,38 (1,97-2,65)	1,08 (1,02-1,13)
Sul	5.038 (31,1)	286 (15,6)	Referência	
Total	16.177 (100,00)	1.828 (100,00)		

IC95%: intervalo de 95% de confiança; OR: *odds ratio*.

Fonte: sistema Gerenciador de Ambiente Laboratorial (GAL).

## Discussão

A implantação-piloto de testes de biologia molecular para detecção de *C. trachomatis* e *N. gonorrhoeae* no âmbito do SUS realizou o aproveitamento da capacidade instalada da rede de laboratórios de saúde pública no país, com compartilhamento dos equipamentos disponíveis para a execução dos exames de quantificação da carga viral de HIV e das hepatites virais B e C distribuídos em todas as 27 UF, sem impactar negativamente a rotina de testagem. Além dos equipamentos, a rede-piloto compartilhou também, em muitos casos, os recursos humanos do laboratório para a execução dos testes, a estrutura logística de envio de insumos aos laboratórios e a rede local de transporte de amostras biológicas, além de possibilitar o acesso aos testes de *C. trachomatis* e *N. gonorrhoeae* às PVHA atendidas pela rede de carga viral de HIV, e que fizeram parte do grupo prioritário de implantação-piloto de *C. trachomatis* e *N. gonorrhoeae*. A estratégia DNO (*diagnostic network optimization*) apoiada pela Fundação para Inovação e Novos Diagnósticos (FIND; Foundation for Innovative and New Diagnostics) visa a promoção do acesso ao diagnóstico de maneira custo-efetiva, e reforça principalmente a importância do compartilhamento da estrutura local para diferentes agravos, como ocorreu na Zâmbia com as redes de HIV e tuberculose ^10^ ^,^ ^11^.

A implantação de uma nova tecnologia na rede de saúde partindo de um estudo-piloto é uma prática comum na área de IST no Brasil ^12^ ^,^ ^13^ ^,^ ^14^, sendo também reconhecida mundialmente como uma iniciativa eficaz para avaliar o desempenho dessa nova tecnologia na população local, identificar as fortalezas do processo e conhecer as barreiras que necessitam ser superadas, visando o sucesso da implantação definitiva ^15^ ^,^ ^16^ ^,^ ^17^ ^,^ ^18^.

A partir das respostas aos questionários aplicados aos profissionais de vigilância da gestão estadual e dos laboratórios, é possível estabelecer estratégias visando a implantação exitosa de uma rede definitiva para detecção de *C. trachomatis* e *N. gonorrhoeae* considerando essa experiência-piloto, como: ampliação da capilarização do acesso à testagem no território e apoio institucional para definição de novos fluxos locais; aumento da sensibilização dos profissionais da atenção à saúde quanto à relevância dos testes de biologia molecular para o manejo clínico dos casos; oferta de *webinars* em horários distintos e/ou disponibilização de gravações, visando ampliar o acesso à informação; disponibilização, pelo nível central, de todos os insumos necessários para a realização dos testes, incluindo o tubo primário de coleta; possibilidade da análise de amostras de diferentes regiões anatômicas com relevância clínica, incluindo amostras menos invasivas (p.ex.: urina), que não demandam estruturas complexas nos serviços de saúde nem profissionais especializados; utilização de kits diagnósticos cujos componentes possibilitem o armazenamento de amostras por longos períodos em temperatura ambiente.

Considerando que existe um alerta mundial devido à crescente taxa de IST causadas por cepas de *N. gonorrhoeae* resistentes aos antimicrobianos ^4^ ^,^ ^5^ ^,^ ^6^, destaca-se que na implantação da rede-piloto, que teve como público-alvo pessoas com maior vulnerabilidade às IST, a proporção de detecção de *N. gonorrhoeae* em mulheres foi de 1,6%, e em homens, de 8,6%. Esses números são superiores à prevalência estimada para a população geral do Brasil, que corresponde a 0,7% (mulheres) e 0,6% (homens) ^19^, e também para a região das Américas, com 0,6% em mulheres e 0,5% em homens ^20^.

O tipo de amostra que apresentou mais associação com a presença de alguma infecção (*C. trachomatis* e/ou *N. gonorrhoeae*) foi a de secreção uretral masculina, mesmo quando comparada com urina masculina - o que era esperado, pois o corrimento uretral é um sintoma característico de infecção por *C. trachomatis* e/ou *N. gonorrhoeae* em homens, com prevalência média de 69% para os casos sintomáticos ^2^. O fato de a positividade ser maior em amostras de corrimento uretral não está relacionado ao desempenho do teste para este tipo de amostra, e sim à maior chance de presença de infecção por *C. trachomatis* e/ou *N. gonorrhoeae* quando há corrimento uretral masculino presente. Portanto, salienta-se que, na prática clínica de testagem em homens, a amostra de urina tem grande utilidade em saúde pública, por ser de fácil coleta e apresentar desempenho semelhante ao do corrimento uretral masculino, quando há presença de infecção. Como não provém de coleta invasiva, a urina é útil tanto para a investigação de infecção nos casos sintomáticos como para fins de rastreio nos assintomáticos, o que pode justificar a menor proporção de amostras positivas de urina quando comparada às amostras de corrimento uretral masculino ^21^ ^,^ ^22^.

Considerando a população alcançada pela rede-piloto, observou-se uma proporção maior de presença de algum tipo de infecção (*C. trachomatis* e/ou *N. gonorrhoeae*) em pessoas mais jovens (≤ 24 anos), do sexo masculino e que se autodeclararam pretas/pardas. Um estudo conduzido por Barbosa et al. ^23^ em homens atendidos em clínicas de IST também encontrou maior prevalência na população de adultos jovens e adolescentes (15-24 anos), considerada pela Organização Mundial da Saúde (OMS) como umas das mais vulnerabilizadas às IST ^20^. No Brasil, as pessoas pretas e pardas ainda encontram importantes barreiras de acesso à prevenção e promoção à saúde ^24^ ^,^ ^25^, apresentando maiores taxas de IST. Dados de notificação nacional revelaram que, em 2021, 51,4% dos casos de sífilis e 63,2% dos casos de HIV ocorreram em pessoas pardas/pretas. Esses dados reforçam a importância de estratégias e ações em saúde pública específicas para atender às necessidades de saúde sexual e reprodutiva dessa população no país.

O estudo apresenta limitações, pois a forma de participação dos serviços na estratégia da rede-piloto foi voluntária; portanto, apesar de a tecnologia estar disponível para laboratórios distribuídos em todas as UF, houve registro de coleta de amostras em 20 delas. Ainda assim, embora não se trate da totalidade do país, considerou-se que a rede-piloto teve boa capilaridade no território nacional, conferindo robustez aos dados analisados. Devido ao banco de dados disponível, não foi possível estratificar as proporções de detecção de *C. trachomatis* e/ou *N. gonorrhoeae* para cada uma das populações consideradas prioritárias na estratégia, tampouco analisar fatores como prática sexual e sintomatologia, que são importantes quando se trata de IST. No entanto, o estudo apresenta, de maneira inédita, dados da implantação de uma oferta de serviço que, até o momento, não estava disponível para a totalidade do país, oportunizando a obtenção de informações para a vigilância de agravos que não são de notificação compulsória no Brasil.

Considerou-se estratégica a aquisição e distribuição dos testes de forma centralizada pelo Ministério da Saúde, o que foi apontado como uma fortaleza pelos gestores locais. Apesar de o resultado não ser liberado no mesmo momento da consulta, destaca-se a importância da realização da testagem não somente para o rastreamento de casos assintomáticos, como também para os casos em que houver a instituição do tratamento de acordo com a síndrome. Isso porque, a partir da visualização do resultado da testagem na consulta de retorno, será possível avaliar melhor o caso, definir o encaminhamento e dar orientação quanto a parcerias sexuais, conforme previsto em protocolo nacional ^2^ ^,^ ^3^ ^,^ ^8^ ^,^ ^26^.

A implantação-piloto de testes moleculares laboratoriais para detecção de *C. trachomatis* e *N. gonorrhoeae* soma-se aos esforços para a melhoria do diagnóstico etiológico e o enfrentamento das IST no país. Os resultados deste estudo demonstraram a importância da disponibilização da testagem em âmbito nacional, sobretudo para as populações com maior vulnerabilidade às IST. As lições aprendidas na implantação-piloto contribuíram para a implantação da rede definitiva para detecção de *C. trachomatis* e *N. gonorrhoeae* por biologia molecular no SUS, considerando a possibilidade de compartilhamento dos equipamentos para quantificação da carga viral de HIV e hepatites virais B e C. Como perspectivas futuras, novos estudos-piloto serão necessários para a implantação de testes moleculares para detecção dos demais patógenos causadores de IST, como *Mycoplasma genitalium* e *Trichomonas vaginalis*, assim como de testes que preveem a resistência além da detecção, e de testes moleculares *point-of-care* para IST realizados fora do ambiente laboratorial, o que permite a liberação do resultado no momento da consulta.
